# Narrow and Stable Single Photon Emission from Dibenzoterrylene in *para*‐Terphenyl Nanocrystals

**DOI:** 10.1002/cphc.202100809

**Published:** 2022-01-12

**Authors:** Ross C. Schofield, Paul Burdekin, Anastasios Fasoulakis, Louise Devanz, Dominika P. Bogusz, Rowan A. Hoggarth, Kyle D. Major, Alex S. Clark

**Affiliations:** ^1^ Centre for Cold Matter Blackett Laboratory Imperial College London Prince Consort Road SW7 2AZ London United Kingdom; ^2^ Quantum Engineering Technology Labs H. H. Wills Physics Laboratory and Department of Electrical and Electronic Engineering University of Bristol BS8 1FD Bristol United Kingdom

**Keywords:** fluorescence spectrosopy, host-guest systems, laser spectroscopy, nanotechnology, single-molecule studies

## Abstract

Single organic molecules are promising photon sources for quantum technologies. In this work we show photon emission from dibenzoterrylene, a widely used organic emitter, in a new host matrix, *para*‐terphenyl. We present a reprecipitation growth method that produces *para*‐terphenyl nanocrystals which are ideal for integration into nanophotonic devices due to their small size. We characterise the optical properties of dibenzoterrylene in nanocrystals at room and cryogenic temperatures, showing bright, narrow emission from a single molecule. Spectral data on the vibrational energies is presented and a further 25 additional molecules are characterised. This emitter‐host combination has potential for quantum technology purposes with wavelengths suitable for interfacing with quantum memories.

## Graphical abstract

**The efficient generation of single photons** for quantum technology is an area of high interest. This work presents a novel single organic molecule emitter and host combination, dibenzoterrylene (DBT) in *para*‐terphenyl. The electronic and vibrational transition energies of DBT in this environment are measured and bright, stable and narrow single photon emission is demonstrated for 26 separate DBT molecules at cryogenic temperatures.

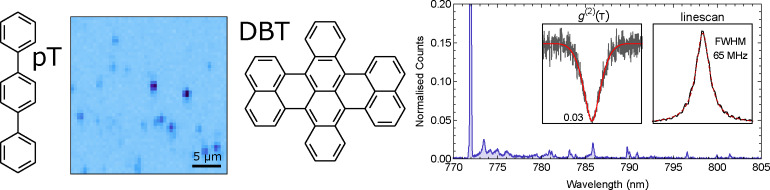

## Introduction

Single organic molecules in the solid‐state have been receiving recent attention for use in quantum devices due to their excellent optical properties and versatility.^[1]^ A number of polycyclic aromatic hydrocarbon (PAH) molecules have been shown to emit photons with a narrow frequency bandwidth which is only limited by their excited state lifetime, making them excellent sources of indistinguishable photons.[^2^, ^3^, ^4^, ^5^] The high photostability and solid‐state nature of these molecules makes them suitable for integration into a wide range of photonic structures,^[6]^ such as optical waveguides[^7^, ^8^, ^9^] and cavities,[^10^, ^11^] which can be used to enhance their interaction with light and enable efficient collection of the photons that they emit. Due to the small size of these molecules, they can also be used as sensitive probes of their local environment, enabling optical readout of changes in electric field,^[12]^ displacement of nearby two dimensional materials,^[13]^ pressure^[14]^ and strain.^[15]^

The combination of emitter and solid‐state host matrix give rise to the properties of the emitted photons,^[16]^ including emission wavelength, homogeneous and inhomogeneous linewidth, and spectral stability. In this work we present a new emitter‐host combination – dibenzoterrylene (DBT) molecules embedded in *para*‐terphenyl (pT) crystals. DBT is a PAH molecule capable of emitting indistinguishable photons,[^4^, ^5^] and is most commonly used in an anthracene (Ac) host matrix where it emits in the near infrared spectral region between 780 nm and 795 nm.^[17]^ pT is a small aromatic molecule that was used as a host matrix in the first experiments to measure fluorescence from a single molecule (pentacene).^[18]^ Since then pentacene in pT has been used for numerous pioneering single molecule studies.^[19]^ In addition, pT has been used as the host for other PAH molecules, such as terrylene.[^20^, ^21^]

Here we exploit the similar solubility of pT and Ac in acetone and water to use a re‐precipitation technique,^[22]^ initially developed for Ac, to grow pT nanocrystals. We first characterise the nanocrystals at room temperature to verify the presence of DBT and confirm the emission wavelength of DBT when embedded in pT, where we find the centre of the inhomogeneous distribution at 772 nm. We then present studies of the coherence properties of DBT molecules in pT at cryogenic temperature.[^23^, ^24^] Fluorescence excitation measurements on a single DBT molecule show a narrow linewidth transition close to the Fourier limit which broadens and saturates as the excitation laser power is increased. By dispersing the fluorescence in a spectrometer we identify the energies of the vibronic states of DBT in pT and this is compared to measurements of DBT in Ac and calculations of DBT vibrational modes in free space.

We analyse fluorescence excitation spectra of a further 25 DBT molecules, showing an inhomogeneous broadening of several nm for stable single molecule emission, typical for DBT in other PAHs.^[25]^ The observed emission wavelength of DBT in pT is compatible with atomic quantum technologies based on potassium ($4S_{1/2}↔4P_{1/2}$ transition) and rubidium $(4P_{3/2}↔5D_{3/2}$ transition). We also report emission at other wavelengths from DBT‐doped pT nanocrystals, which could be from other unstable insertion sites of DBT in the pT crystal structure, or from DBT that is close to the surface of a crystal. The pT nanocrystals are robust to sublimation and can be filtered to select a desired nanocrystal size for particular applications. The small size of these nanocrystals makes them ideal for integration into nanophotonic structures, either directly^[26]^ or after protection in a polymer shell,^[25]^ enabling their use in future photonic quantum devices.

## Results and Discussion

### Room Temperature Characterisation

**Figure 1:**
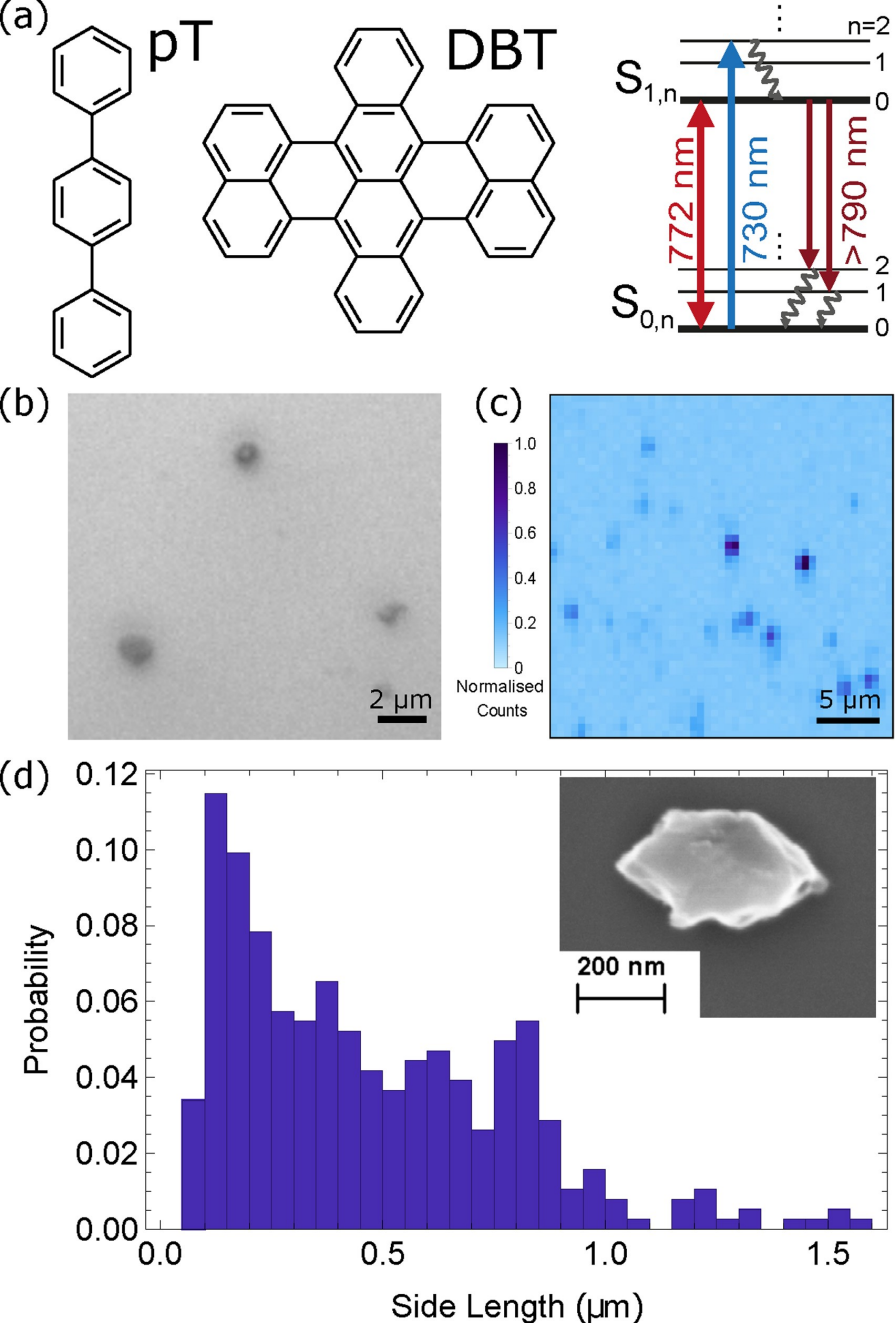
(a) Chemical structures of DBT and pT, with a Jablonski diagram for a DBT molecule within a pT nanocrystal. The molecule can be either be resonantly excited from *S*_0,0_ *→S*_1,0_ on the zero‐phonon line (ZPL, red double‐ended arrow) or off‐resonantly from *S*_0,0_ *→S*_1,*n*>0_ (blue upward arrow), where a fast (picosecond) non‐radiative decay process will occur from *S*_1,*n*>0_ *→S*_1,0_ (curvy black arrow). It will then decay either giving fluorescence on the ZPL, or to vibrational levels of the ground state giving red‐shifted fluorescence (dark red down arrows) where it can then non‐radiatively decay back to the pure electronic ground state. (b) White light microscope image of pT nanocrystals. (c) Spatial fluorescence scan across a sample of DBT‐doped pT nanocrystals showing localised fluorescence. Darker spots correspond to higher photon count rate. Molecules are excited with a 730 nm laser on a *S*_0,0_ *→S*_1,*n*>0_ transition whilst collecting all fluorescence. (d) Size distribution of unfiltered pT nanocrystals. Size determined from side length taken from SEM images. Distribution can be further tailored using filtering after growth.[^22^, ^25^] Inset: Close up SEM image of a single nanocrystal.

We employed a reprecipitation growth method to grow pT nanocrystals in solution.^[22]^ After growth a droplet of this solution was deposited onto a silicon nitride on silica on silicon substrate and allowed to evaporate, leaving behind nanocrystals as shown in the white light microscopy image in Figure 1(b). Identical growth conditions to Ac nanocrystals were used,^[25]^ apart from an increase in growth time from 30 min to 45 min. We determined the size of nanocrystals after growth by analysing scanning electron microscope (SEM) images. A histogram of measured side length is shown in Figure 1(d). Modelling this as a Weibull distribution we find a mean side length of 490 nm with a standard deviation of 350 nm. Greater size selectivity can be achieved by filtering the solution after growth using syringe filters (VWR) which also remove any aggregates. To prevent sublimation of the pT, we coated the samples with a 150 nm thick layer of polyvinyl alcohol (PVA).

We confirmed the presence of DBT inside the pT nanocrystals by putting the sample into a room temperature confocal microscope. We illuminated the molecules with a continuous wave (cw) laser at 730 nm which excites DBT from the ground state to a higher‐lying vibrational level of the excited state (*S*_1,*n*_), shown as a blue arrow in the energy level diagram in Figure 1(a). From here the molecule decays non‐radiatively to the lowest vibrational electronic excited state (*S*_1,0_) in a few picoseconds, before decaying to the ground state and emitting fluorescence (*S*_0,*n*_). The sample was raster scanned using galvanometric mirrors whilst monitoring the collected fluorescence on an avalanche photodiode (APD) after it had passed through a 750 nm long‐pass filter to reject any excitation light. The spatial fluorescence map that results from the scan is shown in Figure 1(c), where many bright spots are visible. Due to the high doping used we expect each nanocrystal, and therefore location, to contain many molecules.

**Figure 2:**
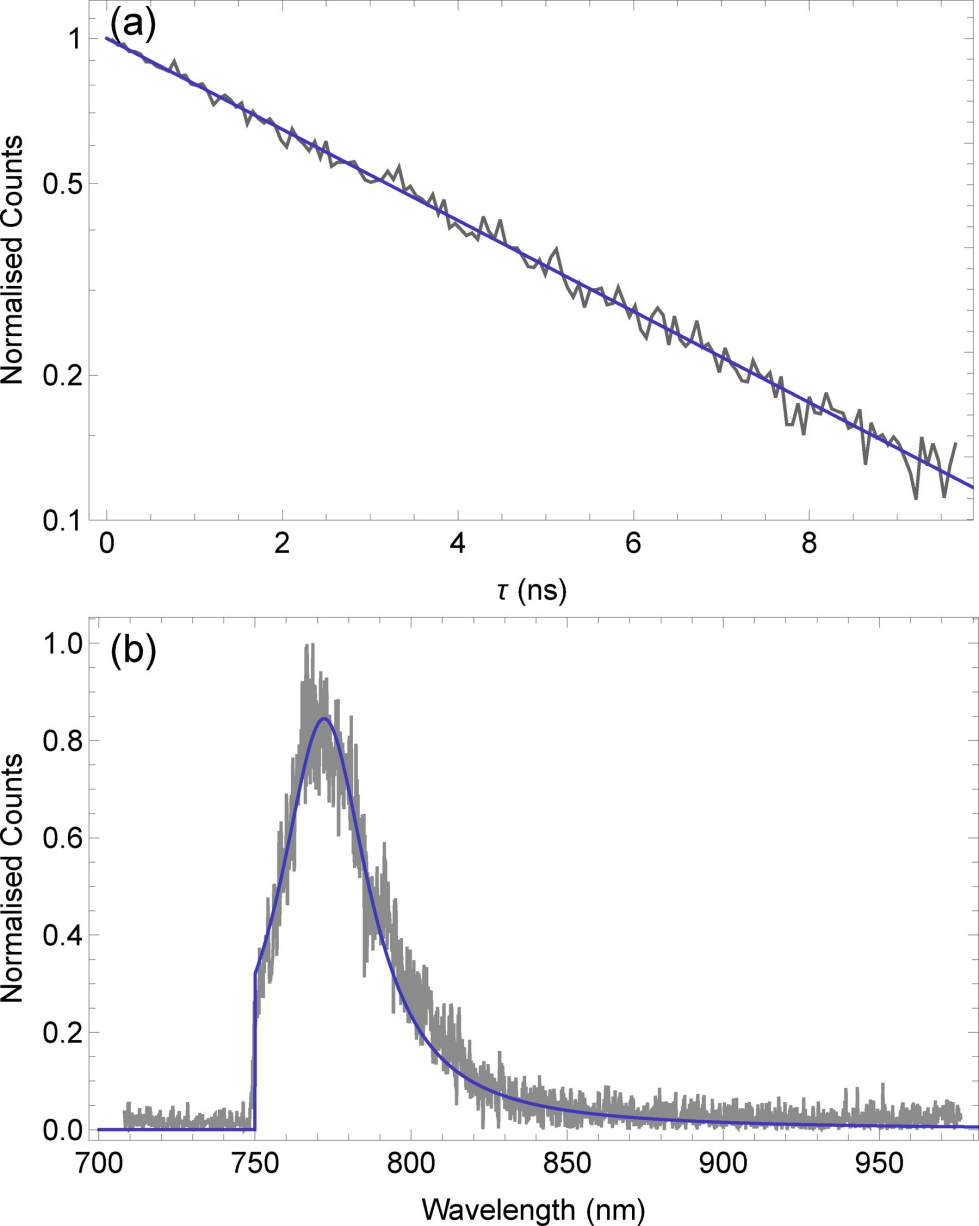
Room temperature measurements on many molecules. (a) TCSPC measurement giving the normalised count rate as a function of time after DBT molecules are excited with a laser pulse at 730 nm. Data is in black, and fitting with an exponential decay, in blue, gives an emitter lifetime of 4.60±0.02 ns. (b) Emission spectrum from many DBT molecules is shown in black. The sharp edge at 750 nm is due to a long‐pass filter. A Lorentzian is multiplied with a step function for the filter and fit to the spectrum, shown in blue, to find a central frequency of 772.1±0.2 nm. This does not account for red‐shifted emission from the phonon‐side band or decay to vibrational states, *S*_1,0_ *→S*_0,*n*>0_.

We excited a bright spot in the confocal scan with 1 mW of pulsed laser at 730 nm and performed a time‐correlated single photon counting (TCSPC) experiment by measuring the time delay between the laser pulse and APD photon detection. A histogram of these times is shown in Figure 2(a). Fitting this data with a single exponential decay we find an excited state lifetime of 4.60±0.02 ns. This is in line with the lifetime of DBT in Ac.[^1^, ^27^] Switching back to the cw excitation, we sent the resulting fluorescence to a spectrometer to obtain a fluorescence spectrum for an ensemble of DBT in pT at room temperature, shown in Figure 2(b). The sharp cut‐on at 750 nm is due to the filter used to reject excitation light. Fitting the spectrum with a single Lorentzian, combined with a step function to account for the filter, gave a central wavelength of 772.1±0.2 nm and a linewidth of ≈9 THz, similar to the 8 THz seen for DBT in Ac at room temperature.^[27]^ This Lorentzian fit does not account for the inhomogeneous distribution of molecules or the vibrational transitions and is intended to be a guide to cryogenic measurements.

### Cryogenic Characterisation

To isolate single molecules and generate narrow bandwidth, coherent photons for quantum information purposes the sample needs to be cooled to cryogenic temperatures. This reduces the phonon population in the solid‐state matrix responsible for thermal dephasing, which is the primary source of photon broadening.^[24]^ We deposited 5 μl of nanocrystals in solution through a 1200 nm pore size syringe filter onto a piece of silicon which had been coated with gold and silica to act as a back reflector. We then covered the nanocrystals with a layer of PVA after evaporation of water as above. This was then placed into a closed‐cycle cryostat and cooled to 5 K.

**Figure 3:**
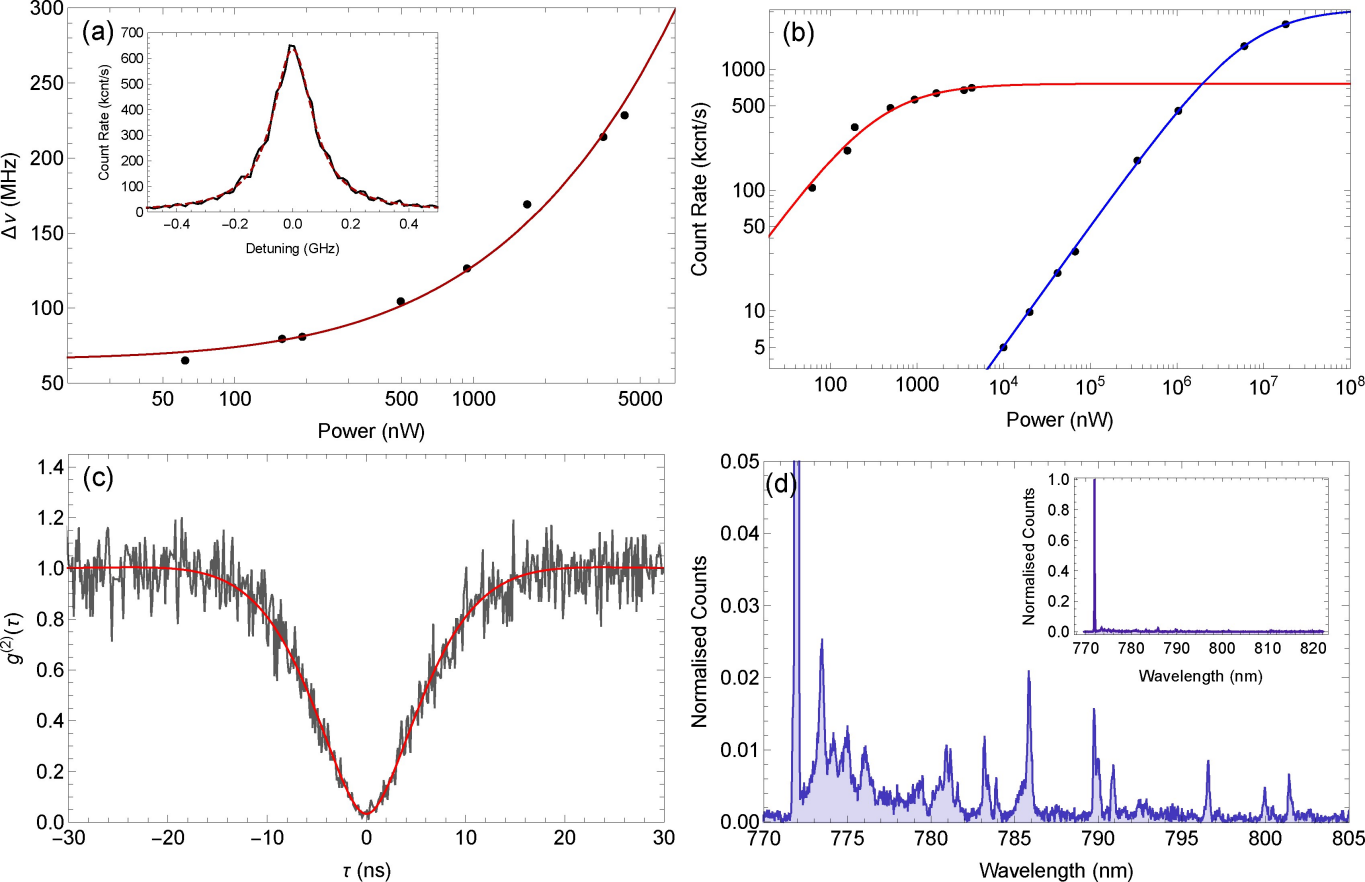
Cryogenic optical properties of a single molecule. (a) Data points are measured linewidth as a function of pump power taken from fitting Lorentzian functions of Eq. 1 to lines seen in the fluorescence excitation spectrum. Fitted line is of the form given in Eq. 2, giving a zero‐power linewidth of 65±4 MHz. Inset: Lorentzian line shape of the red‐shifted fluorescence of the single molecule as a function of laser frequency detuning from the DBT *S*_0,0_ *→S*_1,0_ transition with an excitation power of 1680 nW. (b) Data points are the photon count rate from the molecule using resonant (772.1 nm, data with red line) and blue‐detuned (745.7 nm, data with blue line) excitation at different excitation powers. Fitted lines are of the form given in Eq. 3, giving a maximum resonant count rate of 760±30 kcnt s^−1^ and a maximum blue‐detuned count rate of 3280±8 kcnt s^−1^. (c) Second‐order correlation function measurement showing the number of coincidences with a given delay between detection events taken with 120 nW excitation power. Black is data and red is from fitting with Eq. 4, giving a visibility of *ν*=0.97±0.02 %. (d) Fluorescence spectrum of the molecule pumping at 745.7 nm to excite a *S*_0,0_ *→S*_1,*n*>0_ transition. Data is background subtracted and normalised to the height of the ZPL at 772.05 nm. Note the y‐scale; spectrum is zoomed in to show photon sideband and vibrational transitions. Inset: The full spectrum.

After locating nanocrystals we scanned the wavelength of the excitation laser across the expected range of the $S_{0,0}\to S_{1,0}$ transition, or zero‐phonon line (ZPL), of DBT in pT found from room temperature measurements (around 772 nm). The resulting red‐shifted fluorescence from $S_{1,0}\to S_{0,n>0}$ decay was detected on an APD as the laser scanned across the ZPL of a single molecule. The inset of Figure 3(a) shows an example of scanning the laser across the ZPL transition of a molecule at 772.05 nm. The lineshape is described by the Lorentzian function

(1) $$R=R_{\infty }\frac{S}{1+S+{\left(\frac{2\pi \delta }{\Gamma_{2}}\right)}^{2}}\;,$$

where *δ* $=\nu -\nu_{0}$ is the detuning of the laser frequency, *ν*, from the molecule transition frequency, *ν*_0_, and Γ_2_ is the dephasing rate of the transition.^[28]^ *R* is the measured count rate and $R_{\infty }$ is the maximum count rate of the emitter. *S* is the saturation parameter and can be expressed in terms of the ratio of excitation power *p* to saturation power $p_{\mathrm{sat}}$ as $S=p/p_{\mathrm{sat}}$ .

To find $p_{\mathrm{sat}}$ we measured fluorescence excitation spectra at increasing excitation power, from which we can find the power‐dependent linewidth Δ*ν* which follows the power‐broadening equation

(2) $$\Delta \nu =\frac{1}{\pi }\Gamma_{2}\sqrt{1+S}\;.$$

From this we find $p_{\mathrm{sat}}$ =350±54 nW and Δν= 65±4 MHz, from which we can extract the dephasing rate $\Gamma_{2}=2\pi \times$ 33±2 MHz.^[29]^ The maximum count rate found at when the laser is on resonance with the molecule can also be fitted with the saturation equation

(3) $$R=R_{\infty }\frac{S}{1+S},$$

as shown in Figure 3(b), to find a maximum count rate of $R_{\infty }$ =760±30 kcnt s^−1^ and $p_{\mathrm{sat}}$ =340±40 nW, which agrees with the previous measurement within error. Accounting for losses through the microscope after the power measurement and the focal spot size, this corresponds to an intensity of 0.50±0.06 W cm^−2^.

The purity of the emitted single photon state produced by the molecule was then determined by sending the collected fluorescence to a beam splitter and measuring the time delay between detection events on detectors located at the two output ports. This second‐order correlation function, or $g^{\left(2\right)}\left(\tau \right)$ , measurement is shown in Figure 3(c), showing the characteristic anti‐bunching dip around τ =0 ns. The data is fit with

(4) $$g^{\left(2\right)}\left(\tau \right)=1-𝒱\frac{p+q}{2q}e^{-\frac{1}{2}\left(p-q\right)\left|\tau \right|}+𝒱\frac{p-q}{2q}e^{-\frac{1}{2}\left(p+q\right)\left|\tau \right|}\;,$$

where $p=\Gamma_{1}+\Gamma_{2}$ , $q=\sqrt{{\left(\Gamma_{1}-\Gamma_{2}\right)}^{2}-4\Omega^{2}}$ , and $\Omega =\sqrt{\Gamma_{1}\Gamma_{2}S}$ ,^[29]^ with *S* and Γ_2_ known from above. The fitting gives 𝒱=0.97±0.02 and $\Gamma_{1}=2\pi \times$ 40±2 MHz. This Γ_1_ value corresponds to a lifetime of 4.0±0.2 ns.

This molecule is not lifetime‐limited, with the ratio $\Gamma_{1}/2\Gamma_{2}\approx 0.6$ . However previous work with our cryostat has shown thermal dephasing from the 5 K base temperature as the limiting factor.^[24]^ As such, we expect cooling DBT in pT to 3 K will allow the generation of lifetime‐limited photons.

Next we tuned the laser to 745.7 nm to excite a $S_{0,0}\to S_{1,n>0}$ transition. We repeated saturation measurements using spatial, instead of spectral, scans at increasing powers to measure the maximum count rate and subtract any background. This data is shown in Figure 3(b), and when fit with Eq. 3 we find $R_{\infty }$ =3280±8 kcnt s^−1^ and $p_{\mathrm{sat}}$ =6260±40 μW, or 9.38±0.06 kW cm^−2^.

A long‐pass filter was used to collect all fluorescence whilst rejecting the excitation laser in order to record a fluorescence spectrum. The fluorescence was sent to the spectrometer giving the spectrum shown in Figure 3(d). The spectrum is background subtracted and normalised to the height of the ZPL. The spectrum shows a very strong ZPL at 772 nm with a phonon sideband centered around 775 nm and emission from vibrational transitions at longer wavelengths. Comparison to vibrational energy values from DBT in Ac and density‐functional theory calculations^[30]^ on a free space DBT molecule show closes matches for most of the visible transitions when accounting for a solvent shift. This data is shown in Table 1.

**Table 1:** Energies of vibrational transitions in cm^−1^ for DBT in pT and Ac host matricies and free‐space theoretical calculations. DBT in pT values are taken from the spectrum shown in Figure 3(d). The Ac and theoretical values are taken from Ref. [30]. Ag and Bg refer to the symmetry group of the vibrational mode.

Experimental Measurements		Theoretical	
pT	Ac	Ag	Bg
		93	94
			118
150	176	164	
186	202		184
197			
230	233	226	
	252		251
292	296	291	288
310			
335	331	330	
			362
401	411	412	
454	465	465	422
476	488	491	494
	526		523
	539		532
	586	556	584
617	609	621	626
657	640	654	
	673	687	
			710
			731
780	767	779	782
	777	785	795

The spectrum can be integrated to estimate the branching ratio *α*, or proportion of emission of the ZPL vs the total emission: emission on the ZPL, phonon sideband and vibrational transitions. We find $\alpha_{\mathrm{pT}}=0.55$ for DBT in pT by integrating over the ZPL and dividing by the integral of the entire spectrum. The same methodology performed on DBT in Ac gives $\alpha_{\mathrm{Ac}}=0.46$ using data from Ref. [24], over the same frequency range relative to the ZPL. This suggests a more favourable branching ratio for DBT within pT vs Ac. This method does not give the true branching ratio as there are vibrational energy levels with emission further than 50 nm from the ZPL, however their contribution is likely minor. The Debye‐Waller factor $\alpha_{\mathrm{DW}}$ , or the ratio of the strengths of the ZPL vs the ZPL and phonon sideband, can be found through the same method. As the phonon sideband is fully captured in the spectrum this is a more accurate measure. We find $\alpha_{\mathrm{DW},\mathrm{pT}}=0.79\left(3\right)$ for DBT in pT compared to $\alpha_{\mathrm{DW},\mathrm{Ac}}=0.70\left(6\right)$ for DBT in Ac. Although this is a comparison between two individual molecules and not necessarily representative of all DBT in these host matrices, this does still indicate that pT is a promising DBT host for photons generation for quantum information purposes.

**Figure 4:**
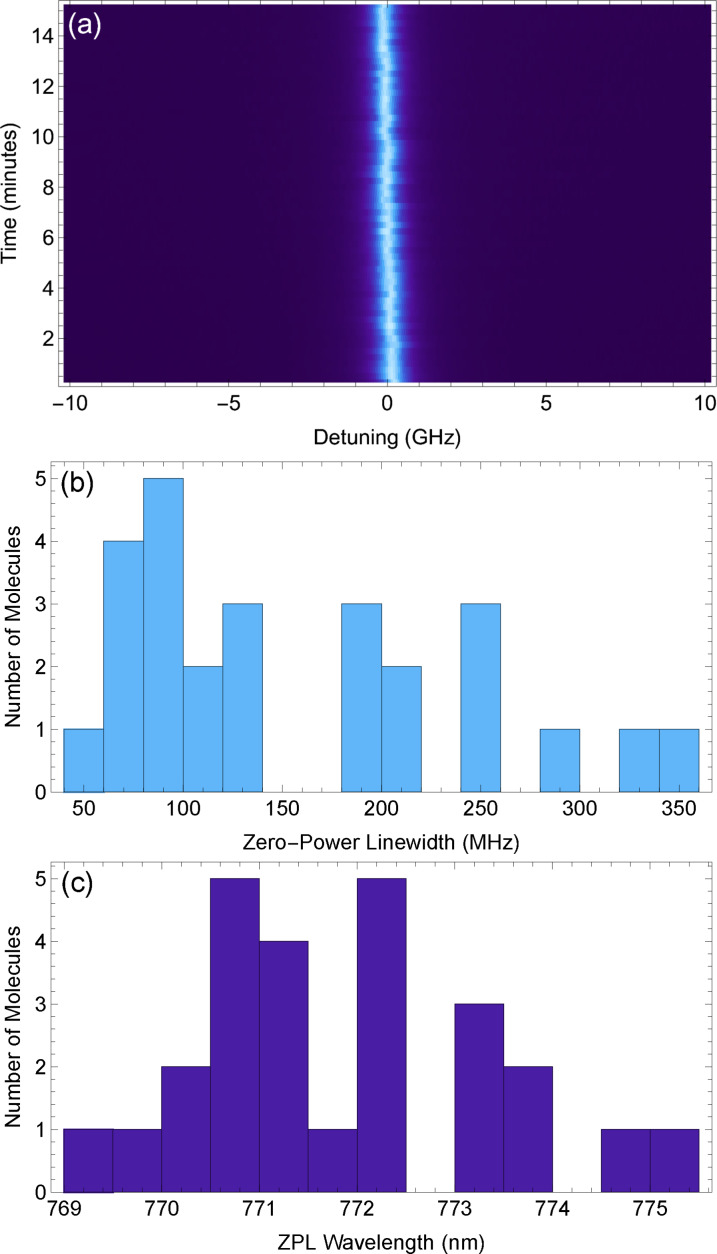
(a) Repeated frequency scans across the *S*_0,0_ *→S*_1,0_ transition of a DBT molecule, showing the long‐term stability of the transition. (b) Histogram of the linewidths of 26 molecules. (c) Histogram of the ZPL transition wavelength of 26 molecules.

We saw no indication of instability during the several hours of constant excitation required for the above measurements, and this is supported by repeated fluorescence excitation spectra measured using a molecule, shown in Figure 4(a). There is only a slight variation ($\ll \Gamma_{2}$ ) in the central frequency of the transition over the 15 minutes of constant scanning. In order to ensure that the molecule shown in Figure 3 was not an anomaly, power‐broadening measurements were repeated for a further 25 molecules. These were found by performing laser scans from 768 nm to 776 nm. The linewidths and wavelengths of the ZPL transition for each of these molecules are shown in Figure 4(b) and (c). 40 % of the molecules display a linewidth Δν< 100 MHz. It is worth noting some nanocrystals scanned contained molecules that bleached almost immediately after excitation, likely due to proximity to the surface of the crystal, however this behaviour is also seen when using Ac as a host material. This data is indicative of a stable and reliable emitter host combination for the generation of narrow single photons.

**Figure 5:**
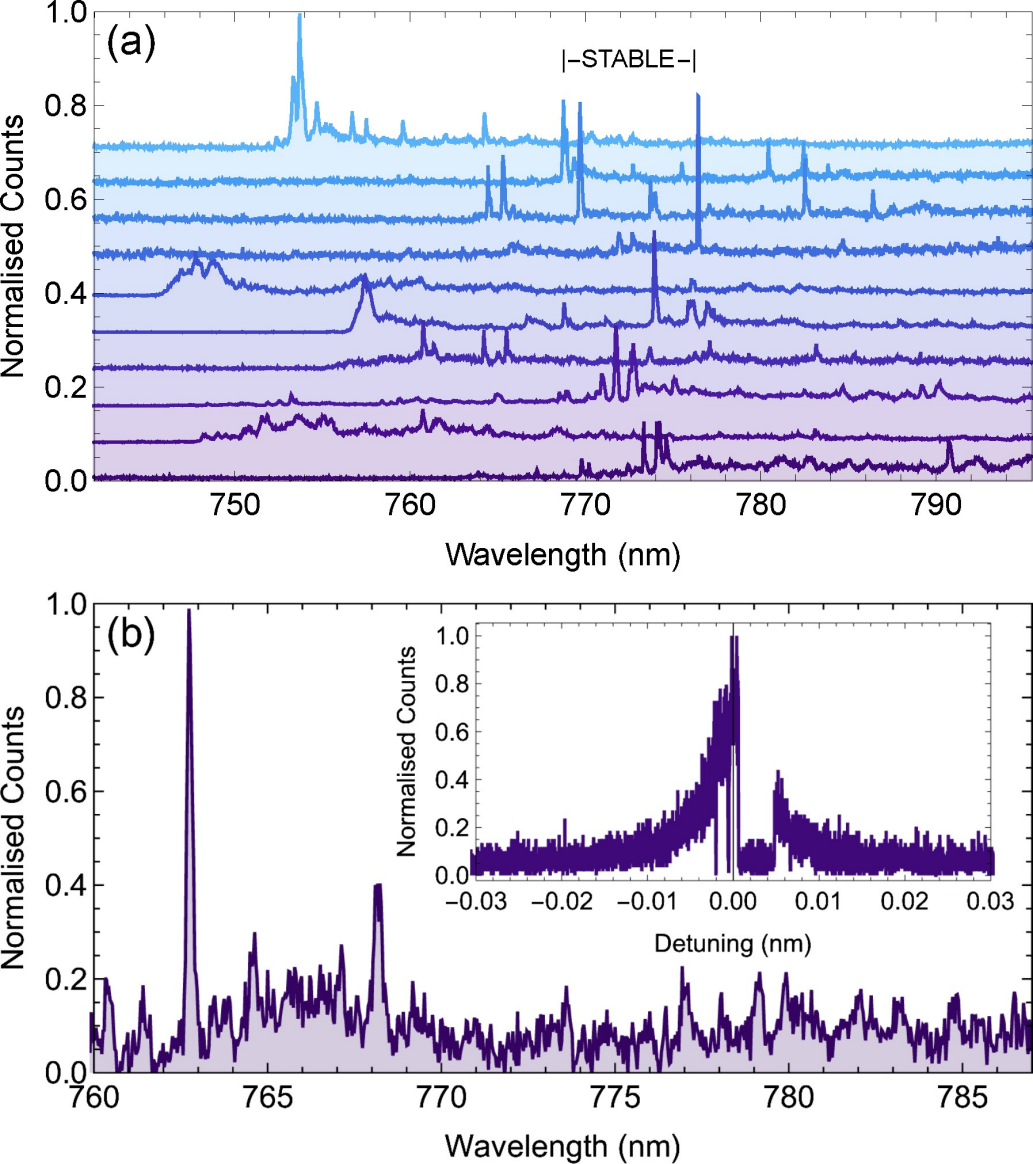
(a) Spectra from different NCs. The approximate wavelength range where stable molecules are found is indicated, 768 nm to 776 nm. (b) Single spectrum showing a ZPL at 762 nm. Inset: Excitation linescan across the ZPL showing spectral instability typical of molecules outside of the stable region. Scan was performed at 2 pm s^−1^.

We also used high power blue‐detuned excitation, at 730 nm to excite any DBT molecules that had ZPL energies outside of the range investigated above. The light from 10 nanocrystals was passed through a 750 nm long‐pass filter tilted to shift the filter edge to ∼745 nm and sent to the spectrometer, giving the spectra shown in Figure 5. There are features that appear similar to ZPL emission outside of the 768 nm to 776 nm range. To investigate if these belonged to alternative insertion sites for DBT molecules, as seen for DBT in other host materials,[^17^, ^31^] we performed laser scans across all features seen in Figure 5. We did not detect any red‐shifted fluorescence from resonant excitation of a majority of these other potential insertion sites. From the features that did show fluorescence, this was unstable over the 10 secs to 30 secs it takes to perform a linescan. Figure 5(b) shows a zoom in on a spectrum with a potential ZPL at 762 nm, however the resonant linescan across this transition shown in the inset shows the spectral instability typical for molecules outside of the identified stable region.

There are a number of reasons why this could be the case. Some of the features which show no fluorescence when scanned in excitation could arise from emission on a transition to an excited vibrational state, $S_{1,0}\to S_{0,n>0}$ . This transition would not be addressable in excitation from *S*_0,0_. Alternatively, the emission could arise from molecules poorly included in the pT nanocrystal or partially embedded in the protective PVA layer that bleach under resonant excitation.

Previous work looking at pentacene^[32]^ and terrylene,^[20]^ molecular single photon emitters similar to DBT, embedded in pT have shown multiple insertion sites with variable spectral stability. In the case of pentacene, there are four different insertion sites depending on which of the four unique pT molecule configurations is replaced. Two of these sites allow the pentacene to remain planar and the other two have significant out‐of‐plane distortions. Although DBT is much larger than pentance and will insert into the pT lattice differently, it is possible the observed spectral lines could be emission from molecules within the pT but in a site that prevents stable emission. The maximum energy difference between the sites in pentacene^[32]^ would correspond to ≈11 nm at 770 nm, which could explain the unstable feature seen in Figure 5(b), for example.

This stability could be influenced by host growth method, with co‐sublimation^[33]^ or melt growth methods^[9]^ being a potential avenue for further research into insertion site stability.

## Conclusions

In this paper we have described a reliable synthesis route for producing DBT containing para‐terphenyl nanocrystals. The size of the nanocrystals and DBT concentration are easy to control during synthesis. Similar nanocrystals have been shown to be suitable for integration into nanophotonic structures.[^1^, ^26^] The comparable physical properties of pT and Ac suggest these techniques will also be suitable for pT. The ease at which the Ac nanocrystal reprecipitation growth method^[22]^ was adapted to pT also highlights its convenience as a method for the development of other aromatic host emitter combinations. The desirable optical properties of DBT are not negatively affected within the pT host matrix when compared to Ac or other materials, and give access to another range of wavelengths suitable for interfacing with other quantum systems. The inhomogeneous broadening spans wavelengths suitable for interfacing with the the potassium D1 line^[34]^ and higher lying transitions in rubidium which are used in ladder‐type quantum memories.^[35]^ We measured many narrow (thermally‐limited) molecules emitting pure single photon states. Preliminary measurements indicate there might be a more favourable ZPL branching ratio for DBT in pT compared to other host matrices, which would be useful for quantum information applications and for coupling DBT to optical cavities.^[11]^ The pT nanocrystals are robust against sublimation, and can be further protected in polymer shells which can allow nanocrystals to be embedded in photonic materials for further processing and integration.^[25]^

## Experimental Section

### Synthesis

**Figure 6:**
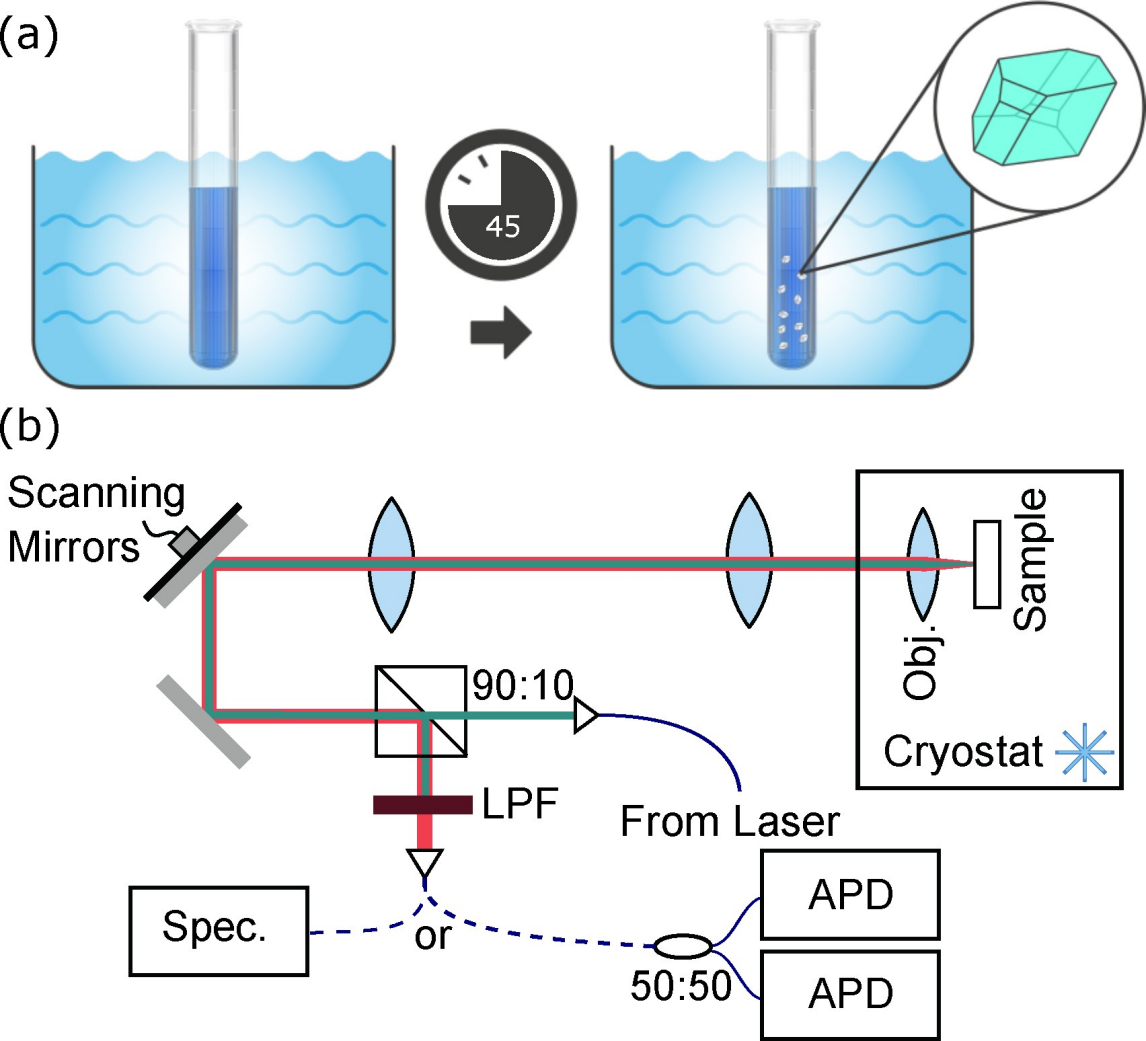
(a) Diagram of the re‐precipitation growth procedure used to prepare the nanocrystals. DBT and pT in acetone are added to 5 ml of sonicated water heated to 50 °C and left for 45 minutes for crystals to form. (b) Schematic of the confocal microscope used for measuring fluorescence from DBT molecules. Dark green is excitation beam path, red is fluorescence collection path, blue is optical fibre. 90 : 10: 90 % reflection:10 % transmission beam splitter; L1: first lens; L2: second lens; Obj.: microscope objective lens; LPF: long‐pass filter; Spec.: spectrometer; 50 : 50: balanced fibre beam splitter; APD: avalanche photodiode

The re‐precipitation method originally detailed in Ref. [22] for Ac nanocrystals was adapted to produce pT nanocrystals. Figure 1(a) shows chemical structures of DBT and pT and Figure 6(a) shows the re‐precipitation growth procedure. 5 ml of distilled water was degassed by sonication for 5 minutes. 10 μl of 10 μmol DBT (MercaChem) in toluene (VWR) solution was added to 10 ml of 5 mmol sublimation‐purified para‐terphenyl (Tokyo Chemical Industry UK) in acetone (VWR) solution. The volume and concentration of DBT in toluene solution added can be varied to change the number of molecules present in the nanocrystals. 250 μl of this mixed solution was then added to the distilled water and sonicated at 37 KHz and 50 °C for 45 minutes. This growth time is longer than the 30 minutes used for Ac nanocrystals,^[25]^ as we found this gave more consistent nanocrystals. The vial was left uncovered for the growth.

The solution can then be passed through a syringe filter to remove larger crystals if desired. A 5 μl droplet was deposited onto a substrate and the water was allowed to evaporate. 10 mm by 10 mm sections of two different substrates were used, either 120 nm silicon nitride (Si_3_N_4_) coated on top of 1 μm of silica (SiO_2_) on a 500 μm thick silicon (Si) wafer, or 85 nm SiO_2_ on Au on a 500 μm thick Si wafer. Both substrates have good thermal conductivity for use in the cryostat, with the Au wafer also being highly reflective to maximise the photon collection efficiency from the DBT molecules. The nanocrystals are then spin‐coated at 4000 rpm with 4 % w/w PVA in water solution, giving an approximate thickness of 150 nm, to protect them from sublimation.

### Microscopy Setup

White light microscopy was performed using a Nikon Eclipse L200. Either 20x (LU Plan 20x, 0.4 NA, Nikon) or 50x (LU Plan 50x, 0.8 NA, Nikon) microscope objective lenses are used with a CCD camera (Hamamatsu C4742‐95). Scanning electron microscopy measurements were taken using a Raith E‐line EBL and SEM system, operating at 10 kEv.

Two confocal microscopes were used to measure the optical properties of DBT molecules within the pT nanocrystals. These are functionally identical apart from the presence of a cryostat on one to allow for cooling to 5 K. Figure 6(b) shows a simplified schematic of the confocal microscope setup. Two tunable laser systems were used, either a continuous‐wave Ti:Sapphire (SolsTiS, MSquared) laser or a pulsed Ti:Sapphire (Tsunami, Spectra Physics) laser, and these were coupled into the microscope and collimated. The excitation beam was spectrally filtered to remove any background contributions from the fibre and a 90 % reflection:10 % transmission beam splitter was used to combine the excitation and collection paths. Scanning mirrors were used in combination with two lenses, L1 and L2, in a ‘4 f’ configuration to allow a change in angle of the scanning mirrors to change the input angle of the excitation beam to the objective lens. This corresponds to a change in focal position on the sample, allowing for raster scanning of the scanning mirrors to produce a fluorescence ‘map’ of the sample as the beam scans across the position of molecules. An example of this is shown in Figure 1(c).

The fluorescence coupled into a multimode fibre was either sent to an avalanche photodiode (APD, Count‐T, Laser Components), a multimode fibre beam splitter (MMF 50 : 50) connected to two APDs for correlation measurements, or a spectrometer (Shamrock 303i with Newton EMCCD, Andor). A timing card (Hydraharp, PicoQuant) was used for any time‐tagging and correlation experiments. For the second‐order correlation function measurement in Figure 3(c) we convolved Eq. 4 with a 320 ps Gaussian to account for detector timing jitter.

## Conflict of interest

The authors declare no conflict of interest.

1

## Data Availability

The data that support the findings of this study are available from the corresponding author upon reasonable request.
